# Advanced lung adenocarcinoma harboring uncommon EGFR 19 Del and T790M/trans-C797S mutations after resistance: a case report and literature review

**DOI:** 10.3389/fonc.2025.1525885

**Published:** 2025-04-16

**Authors:** Yuting Xiao, Dunqiang Ren, Huanhuan Bi, Yinxue Zhou, Yanmei Shao, Weizhong Han, Na Na, Hongmei Wang

**Affiliations:** Department of Respiratory and Critical Care Medicine, The Affiliated Hospital of Qingdao University, Qingdao, China

**Keywords:** lung adenocarcinoma, uncommon EGFR mutations, T790M mutation, trans-C797S, EGFR-TKI resistance, combination therapy, case report, literature review

## Abstract

The most common epidermal growth factor receptor (EGFR) mutation in non-small cell lung cancer (NSCLC) is exon 19 deletion (19del), which is sensitive to EGFR tyrosine kinase inhibitors (EGFR-TKIs). However, uncommon EGFR 19del mutations exhibit varied responses to EGFR-TKI treatment. Research and clinical data on these uncommon subtypes are limited. Additionally, resistance to EGFR-TKIs is inevitable. EGFR C797S is a frequent mechanism of resistance to third-generation EGFR-TKIs, usually occurs in cis with T790M and in 5% of patients in trans. Here, we report a patient diagnosed with lung adenocarcinoma harboring EGFR 19Del L747-A755delinsSKD mutation with co-occurring T790M and trans-C797S mutations, who showed a positive response to combination therapy with first- and third-generation TKIs. This case report suggests an effective treatment option for such patients.

## Introduction

Epidermal growth factor receptor (EGFR) mutations are prevalent driver genes in the pathogenesis and progression of non-small cell lung cancer (NSCLC) (1). Among all EGFR mutations, exon 19 deletion (19del) is the most frequent (45%) and is generally more sensitive to tyrosine kinase inhibitors (TKIs) than other common EGFR mutations (2, 3). However, not all 19del alterations are considered as “golden” mutations. The predominant 19del subtype is E746-A750del (66.1%), followed by delL747-P753insS (9.7%) and L747-T751 (6.9%) (4). Moreover, there are over 70 uncommon subtypes and their response to TKIs is still unclear (5). After first- or second-generation EGFR-TKIs therapy, EGFR T790M is the most common secondary mutation. Although it can be overcome by osimertinib and other third generation inhibitors, resistance seems inevitable as well (6, 7). Resistance to the third-generation EGFR-TKIs can be mainly divided into EGFR-dependent and -independent ones. EGFR-dependent mechanism refers to manifold EGFR mutations while EGFR-independent mechanisms include bypass signal activation, histologic transformation and so on (8). The coexistence of T790M and C797S accounts for approximately 18% of all resistant mutations, with trans-mutations accounting for less than 15% (9). Clinical evidence of EGFR T790M and C797S in trans is still needed. Herein, we present a case report of lung adenocarcinoma harboring the rare EGFR L747_A755delinsSKD mutation. Additionally, EGFR T790M and C797S in trans were detected during treatment. In conjunction with the existing literature on C797S in trans, we conducted a comprehensive analysis to provide real-world data reference for these patients.

## Case presentation

**Figure 1:**
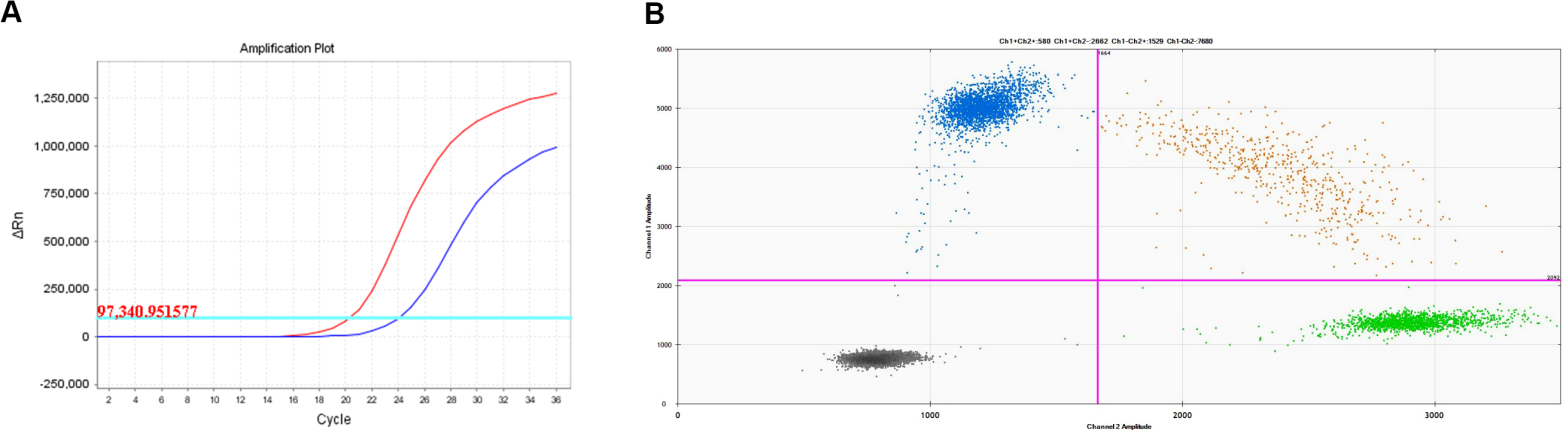
Molecular analysis on lung biopsy. **(A)** EGFR 19 deletion mutation; **(B)** EGFR T790M mutation.

**Figure 2:**
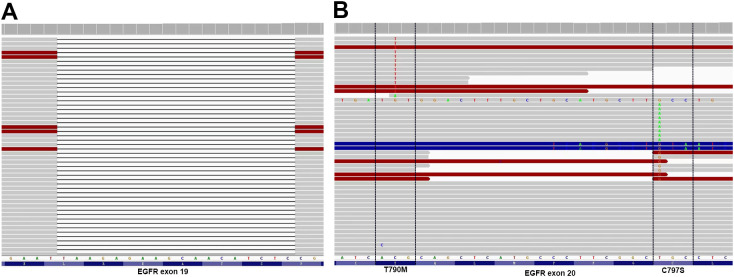
Allelic context on plasma before First- and Third-Generation EGFR TKIs combination therapy. **(A)** EGFR Exon19 alteration (p. L747-A755delinsSKD); **(B)** EGFR C797S located in trans with T790M.

**Figure 3:**
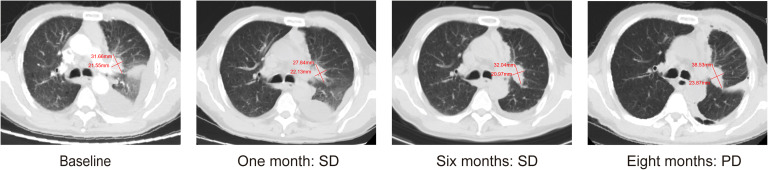
Representative computed tomography images of lung lesions.

**Figure 4:**
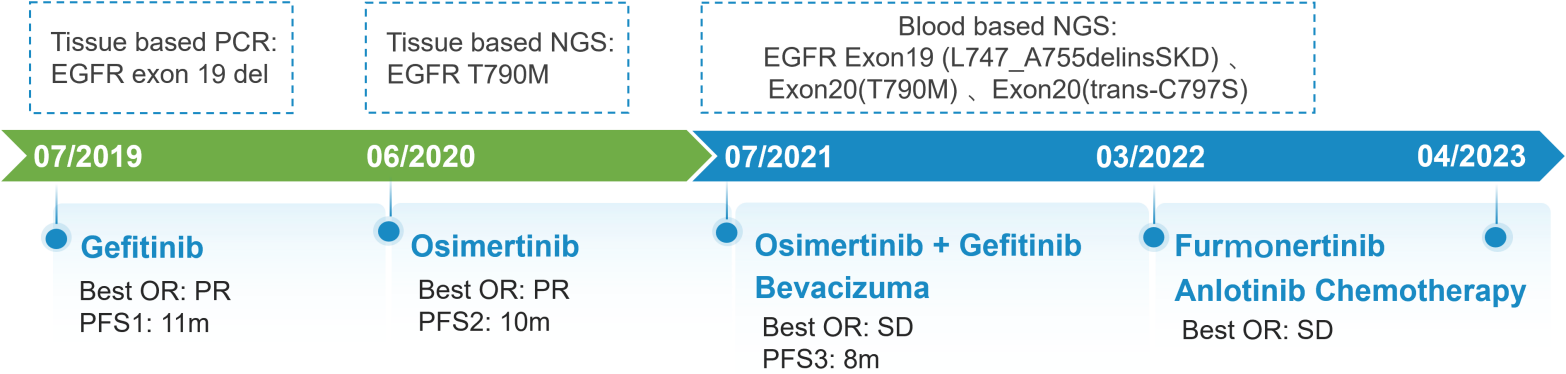
Schematic summary of treatment course. PCR, polymerase chain reaction; EGFR, epidermal growth factor receptor; OR, objective response; PFS, progression free of survival; NGS, next generation sequencing.

A 67-year-old man presented with a persistent dry cough for more than 3 months in July, 2019. The patient had a smoking history of 50 years with 30 cigarettes a day and he denied any other medical or family history. Contrast enhances chest computed tomography (CT) revealed a mass in the central left upper lobe, enlarged bilateral mediastinal lymph nodes and bilateral pulmonary nodules. A biopsy of the lesion under tracheoscopy confirmed lung adenocarcinoma histologically. Macroscopic metastases were observed in the bone by Whole Body Scan (WBS), and the patient was clinically classified as stage IVB (T4N0M1c) NSCLC. Zoledronic acid was administered to control bone destruction during the treatment. Owing to the deletion of EGFR exon 19 detected by Amplification Refractory Mutation System Polymerase Chain Reaction (ARMS-PCR) in the tumor tissue (**Figure 1A**), the patient began treatment with gefitinib 250 mg once daily, resulting in a radiological response and rapid clinical benefit lasting 11 months. Subsequent tissue biopsy at the time of relapse confirmed an acquired T790M in exon 20 (c.2369C>T, frequency as 61.9%) by droplet digital polymerase chain react (ddPCR) (**Figure 1B**). He was then switched to osimertinib 80mg once daily, achieving stable disease with shrinkage of hepatic lesions. Almost 10 months later, the patient was readmitted to the hospital because of malignant pleural effusion. Next generation DNA sequencing (NGS) using a plasma sample was conducted to identify potential targets, revealing an EGFR exon 20 mutation (p.T790M, c.2369C>T, frequency as 0.26%), an EGFR exon 20 mutation (p.C797S, c.2389T>A, frequency as 0.20%, **Figure 2B**), and an EGFR Exon19 alteration (p.L747-A755delinsSKD, c.2240-2264>CGAAAGA, frequency as 1.36%, **Figure 2A**, **Supplementary Table S1**). Based on these NGS results, the patient was started on gefitinib 250mg daily combined with osimertinib 80mg daily as a further-line treatment. Cisplatin and bevacizumab were added to control malignant pleural effusion. The patient experienced a significant improvement in dyspnea, cough, fatigue, and malaise within one week. However, 8 months after starting the combination TKI therapy, dyspnea worsened with evidence of increased left pleural effusion and disease progression in the lungs and lymph nodes (**Figure 3**, **Supplementary Figure S1**). The patient refused further biopsy owing to physical and financial conditions. He was then undergoing bevacizumab and pemetrexed for four cycles but was switched to a regimen of oral anlotinib 8mg and furmonertinib 160mg daily after progression. Again, target therapy resulted in a stable disease lasting five months. Adverse effects, including fatigue and gastrointestinal symptoms, were controlled with supportive care. Because of ongoing clinical benefit, the patient continued receiving anlotinib and furmonertinib for a further 3 months as salvage treatment. However, the patient’s condition continued to deteriorate and died in 2023 (45 months after the diagnosis of lung cancer) (**Figure 4**, **Supplementary Figure S2**).

## Discussion

Exon 19 predominantly harbors a deletion mutation within codons 746-752, resulting in the elimination of four highly conserved amino acids (LREA) from the amino acid sequence (10). Based on the number of deleted bases, initiation codon deletions, and common in-frame deletions, they can be classified into various subtypes (11). Chung et al. reported patients with LRE deletions in exon 19 had a better response to EGFR-TKIs than those with non-LRE deletions (12). Studies disclosed that patients with the exon 19 deletion starting on codon E746 had a better median PFS, compared to those starting on L747 (13, 14). However, Peng et al. indicated that patients with uncommon EGFR 19delins have better clinical outcomes (15). Another study found no statistically significant difference in PFS between the two groups (HR=0.89; P=0.468) (16). These differences may be due to the limited sample sizes of these studies. As for the L747-A755delinsSKD subtype observed in our case, all of the initiation codon, base pair deletion length, and amino acid insertions were uncommon. Studies have also demonstrated that deletions with amino acid insertions have a poorer prognosis compared to deletions without insertions(P=0.0244) (11, 17, 18). Fortunately, the deletion fragment in our patient includes the LREA sequence, which may explain the sensitivity EGFR-TKI drugs (19). Similarly, the reported PFS1 and PFS2 of our patient were shorter than those reported in clinical studies. In conclusion, further *in vitro* and clinical studies are still needed.

As for resistance mechanisms, studies have suggested that the E746-A750del subtype has a higher frequency of acquiring T790M mutation compared to other 19del subtypes (16). However, similarities in resistance patterns have been observed between the E746-initiated and L747-initiated subtypes (15, 16). The tertiary EGFR mutations are responsible for the third-generation EGFR TKIs resistance, in which C797S mutation is the major one. The cysteine is substituted by a less nucleophile serine in the EGFR C797 position, leading to the destruction of a covalent bond between the mutant receptor and inhibitor (8). In previous studies, only one case harboring L747-A755delinsSKD subtype was reported, which also acquired T790M/trans C797S after osimertinib resistance (20). Unfortunately, the patient soon succumbed to disease. The efficacy of 1st and 3rd generation TKIs has been confirmed in *in vivo* studies (21).Durable response have been observed in our patient after the combination therapy. Here, we reviewed previous reports on T790M/C797S in trans. The search strategy is detailed in **Supplementary Table S2** and the study flowchart is provided in **Supplementary Figure S3**. Despite its safety profile, PFS varied from 6 weeks to 14 months among patients (**Table 1**), which is the same as demonstrated in a large retrospective study. Besides, the patients who had durable response to the combination regimen also tended to have better OS. Three factors may be at play in combination therapy: dynamic changes of EGFR C797S-carrying clones, resistance to third-generation TKIs, and whether T790M and C797X are in the same clone (22). Notably, previous investigations have often not mentioned the specific subtype of exon19 deletion. The roles distinct subtypes of exon 19 deletion paly in combination therapy await validation with larger, prospective studies such as the ORCHARD trial (NCT03944772). VEGFR inhibitors has demonstrated favorable therapeutic outcomes in patients with cis C797S/T790M mutations (23, 24). Cases from the table below also revealed the potential advantages for patients harboring the trans-C797S/T790M mutation (25, 26).

**Table 1:** Detailed clinical and molecular characteristics of each patient who acquired T790M and trans-C797S mutation after relapse from EGFR-TKIs therapy.

Year	Author	Patient	Metastasis	Former-line	Gene	Treatments	PFS	OS
2017	Arulananda, Surein et al. (27)	41, Male	Mediastinum, bone	Erlotinib; Osimertinib; Nivolumab	19Del, T790M, trans-C797S	Gefitinib + Osimertinib	6W	26m
2017	Zhen Wang et al. (28)	43, Male	Brain	Afatinib; Osimertinib	19Del, T790M, trans-C797S	Erlotinib + Osimertinib	3m	42m
2019	Zhen Zhou et al. (25)	42, Female	Brain, bone	Erlotinib; Osimertinib	19Del, T790M, trans-C797S	Gefitinib + Osimertinib; Erlotinib + Osimertinib, bevacizumab	8m	58m
2019	Elio G. Pizzutilo et al. (20)	75, female	lungs, MPE, LN, liver, adrenal gland, bone.	Gefitinib; Osimertinib;	19Del (L747-A755delinsSKD), T790M, trans-C797S, SCLC	Carboplatin monotherapy	6W	24m
2020	Xiaoyan Wang et al. (29)	42, Female	LN, thorax, pericardium	Erlotinib; Osimertinib	19Del, T790M, trans-C797S	Erlotinib + Osimertinib	14m	NA
2020	Jianxin Chen et al. (30)	52, Male	Chest wall	Icotinib; Osimertinib; Anlotinib	19Del (E746-A750del), T790M, trans-C797S, CTNNB1	Gefitinib + Osimertinib	1m	64m
2020	Yubo Wang et al. (31)	53, Male	Lung, LN, pericardium	Gefitinib; Osimertinib;	19Del, T790M, trans-C797S	NA	NA	30.5m
2021	Zhou, Rengui et al. (24)	52, Female	Lung	Gefitinib; Osimertinib;	19Del, T790M, trans-C797S	Gefitinib + Osimertinib	8+m	39m
2023	Chen, Yang et al. (26)	58, Male	Lung, bone	Gefitinib; Osimertinib+pemetrexed	19Del, T790M, C797S in trans and cis	Osimertinib + Anlotinib	8m	NA
2024	Rivera-Concepcion, Joel (32)	48, Female	Bone	Erlotinib; Osimertinib; Carboplatin+etoposide+atezolizumab	19 Del, T790M, trans-C797S, RB1 and TP53 LOF mutations	Gefitinib + Osimertinib	3m	60m

EGFR, epithelial growth factor receptor; Del, deletion; NA, not available; PFS, progression-free survival; LN, lymph node; MPE, malignant pleural effusion; SCLC, small cell lung cancer.

This study has certain limitations. Owing to the constraints of the available detection methods at that time, we were unable to identify the exact EGFR 19 subtype during the initial diagnosis. Furthermore, genetic testing was not repeated after the progression of the first- and third-generation TKI combination therapy because of the patient’s body function, family support, and economic conditions. Consequently, our knowledge regarding changes in genes following resistance development remains incomplete. Challenges remain in conducting repeated biopsies and genetic tests, especially in settings with limited resources. A balanced approach is crucial, requiring a comprehensive assessment of the patient’s health, treatment benefits and risks, and personal preferences. In summary, this study emphasized maintaining open communication with patients and striving for the best disease management and quality of life outcomes.

## Conclusion

Briefly, we present a case of uncommon EGFR 19 Del(L747-A755delinsSKD) and T790M/trans-C797S mutations after resistance, showing a positive response to combination therapy with first- and third-generation EGFR-TKIs. Distinct subtypes of exon 19 deletion may influence both trans C797S production and response to combined first- and third-generation TKIs therapy. Angiogenesis inhibitors may further improve the efficacy of EGFR TKIs, and dynamic monitoring of gene mutations is necessary throughout the treatment process. However, further basic experiments and clinical studies are warranted.

## Data Availability

The raw data supporting the conclusions of this article will be made available by the authors, without undue reservation.
