# Supplementary material for: Advanced lung adenocarcinoma harboring uncommon EGFR 19 Del and T790M/trans-C797S mutations after resistance: a case report and literature review
Source: Front Oncol. 2025 Apr 16;15:1525885. doi: 10.3389/fonc.2025.1525885 (PMC12040630; doi:10.3389/fonc.2025.1525885)
Supplement: Supplementary file 1 [file DataSheet1.docx]

**Supplementary material**

**Figure S1. Representative computed tomography images of lung lesions and lymph nodes.**


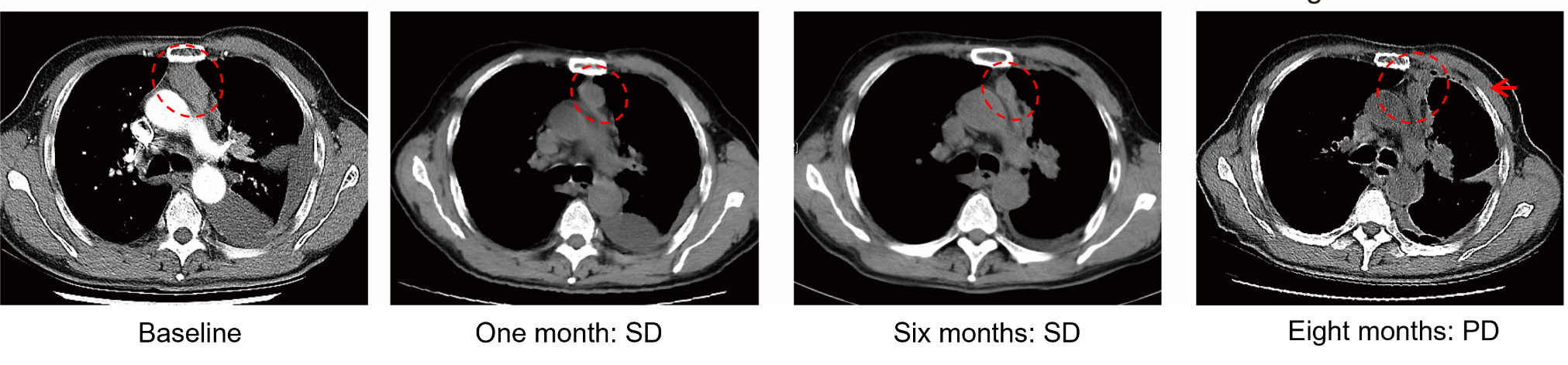


**Figure S2. A schematic of the EGFR mutation evolution over time.**

**
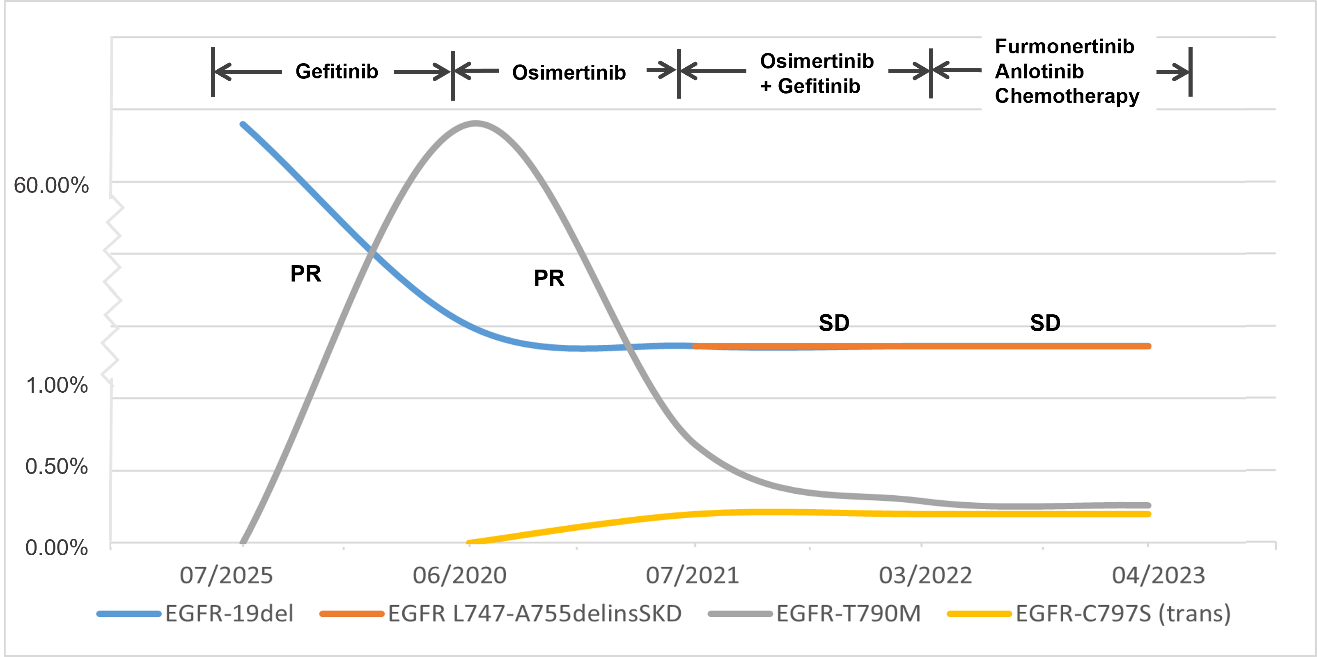
**

**Table S1. The nucleotide variant of the mutation.**

|  | **755** | **746** | **747** | **748** | **749** | **750** | **751** | **752** | **753** |  | **754** | **755** | **756** | **757** | **758** | **759** | **760** | **761** | **762** |
| --- | --- | --- | --- | --- | --- | --- | --- | --- | --- | --- | --- | --- | --- | --- | --- | --- | --- | --- | --- |
| **Wild type** | **K** | **E** | **L** | **R** | **E** | **A** | **T** | **S** | **P** |  | **K** | **A** | **N** | **K** | **E** | **I** | **L** | **D** | **E** |
|  | AAG | GAA | TTA | AGA | GAA | GCA | ACA | TCT | CCG |  | AAA | GCC | AAC | AAG | GAA | ATC | CTC | GAT | GTG |
| **E746-A750** | **K** | **-** | **-** | **-** | **-** | **-** | **T** | **S** | **P** |  | **K** | **A** | **N** | **K** | **E** | **I** | **L** | **D** | **E** |
|  | AA- | --- | --- | --- | --- | --A | ACA | TCT | CCG |  | AAA | GCC | AAC | AAG | GAA | ATC | CTC | GAT | GTG |
| **L747-A755delinsSKD** | **K** | **E** | **S** | **-** | **-** | **-** | **-** | **-** | **-** |  | **K** | **D** | **N** | **K** | **E** | **I** | **L** | **D** | **E** |
|  | AAG | GAA | T -- | --- | --- | --- | --- | --- | -CG |  | AAA | GAC | AAC | AAG | GAA | ATC | CTC | GAT | GTG |

**Table S2. Literature search** **strategy*.**

| **PubMed** | | |
| --- | --- | --- |
| #1 | "lung cancer"[Title/Abstract] OR "lung carcinoma"[Title/Abstract] OR "lung neoplasm"[Title/Abstract] OR "NSCLC"[Title/Abstract] OR "lung adenocarcinoma"[Title/Abstract] | [244,331](https://pubmed.ncbi.nlm.nih.gov/?term=%22lung+cancer%22%5BTitle%2FAbstract%5D+OR+%22lung+carcinoma%22%5BTitle%2FAbstract%5D+OR+%22lung+neoplasm%22%5BTitle%2FAbstract%5D+OR+%22NSCLC%22%5BTitle%2FAbstract%5D+OR+%22lung+adenocarcinoma%22%5BTitle%2FAbstract%5D&sort=relevance&size=200&show_snippets=off&ac=no) |
| #2 | "epidermal growth factor receptor"[Title/Abstract] OR "EGFR"[Title/Abstract] | [109,555](https://pubmed.ncbi.nlm.nih.gov/?term=%22epidermal+growth+factor+receptor%22%5BTitle%2FAbstract%5D+OR+%22EGFR%22%5BTitle%2FAbstract%5D&sort=relevance&size=200&show_snippets=off&ac=no) |
| #3 | "C797S"[Title/Abstract] | [371](https://pubmed.ncbi.nlm.nih.gov/?term=C797S%5BTitle%2FAbstract%5D&sort=relevance&size=200&show_snippets=off&ac=no) |
| #4 | "randomized controlled trial"[Publication Type] OR "clinical trial"[Publication Type] OR "Trial"[Title/Abstract] OR "Randomized"[Title/Abstract] OR "randomised"[Title/Abstract] OR "randomly"[Title/Abstract] OR "phase"[Title/Abstract] OR "case reports"[Publication Type] OR "Comment"[Publication Type] OR "Case"[Title/Abstract] | [7,609,392](https://pubmed.ncbi.nlm.nih.gov/?term=%22randomized+controlled+trial%22%5BPublication+Type%5D+OR+%22clinical+trial%22%5BPublication+Type%5D+OR+%22Trial%22%5BTitle%2FAbstract%5D+OR+%22Randomized%22%5BTitle%2FAbstract%5D+OR+%22randomised%22%5BTitle%2FAbstract%5D+OR+%22randomly%22%5BTitle%2FAbstract%5D+OR+%22phase%22%5BTitle%2FAbstract%5D+OR+%22case+reports%22%5BPublication+Type%5D+OR+%22Comment%22%5BPublication+Type%5D+OR+%22Case%22%5BTitle%2FAbstract%5D&sort=relevance&size=200&show_snippets=off&ac=no) |
| #5 | 0001/01/01:2024/05/01[Date - Publication] | [37,187,904](https://pubmed.ncbi.nlm.nih.gov/?term=%28%220001%2F01%2F01%22%5BDate+-+Publication%5D+%3A+%222024%2F05%2F01%22%5BDate+-+Publication%5D%29&sort=relevance&size=200&show_snippets=off&ac=no) |
| #6 | #1 AND #2 AND #3 AND #4 AND #5 | [99](https://pubmed.ncbi.nlm.nih.gov/?term=%231+AND+%232+AND+%233+AND+%234+AND+%235&sort=relevance&size=200&show_snippets=off&ac=no) |
| **Web of Science** | | |
| #1 | TS=(“lung cancer” OR “lung carcinoma” OR “lung neoplasm” OR ”NSCLC” OR “lung adenocarcinoma”)  Timespan=All years | [539,350](https://webofscience.clarivate.cn/wos/alldb/summary/7d2ac1d2-931c-49a8-8b30-7e1ed217691b-e96256b3/relevance/1) |
| #2 | TS=(“epidermal growth factor receptor” OR EGFR)  Timespan=All years | [182,497](https://webofscience.clarivate.cn/wos/alldb/summary/30368354-ad72-43b5-817e-2fae4dd777be-e9626495/relevance/1) |
| #3 | TS=(“C797S” )  Timespan=All years | [559](https://webofscience.clarivate.cn/wos/alldb/summary/1c8012b4-0d31-4448-ba2f-cd9d4d94a659-e9626942/relevance/1) |
| #4 | TS=(random* OR “trial”OR “phase”OR” case”OR” comment” )  Timespan=All years | [4,294,868](https://webofscience.clarivate.cn/wos/alldb/summary/3523bf81-bb5a-46b9-b86c-b1b408372a32-e962744c/relevance/1) |
| #5 | #1 AND #2 AND #3 AND #4 | [33](https://webofscience.clarivate.cn/wos/alldb/summary/71d88396-83d0-493c-bab1-137b9ea1f7bf-e9627a3c/relevance/1) |
| **Embase** | | |
| #1 | 'lung cancer':ti,ab,kw OR 'lung carcinoma':ti,ab,kw OR 'lung neoplasm':ti,ab,kw OR nsclc:ti,ab,kw OR 'lung adenocarcinoma':ti,ab,kw | 377797 |
| #2 | 'epidermal growth factor receptor':ti,ab,kw OR egfr:ti,ab,kw | 193974 |
| #3 | c797s:ti,ab,kw | 729 |
| #4 | 'controlled clinical trial':it OR 'randomized controlled trial':it OR 'case reports':it OR comment:it OR trial:ti,ab,kw OR randomized:ti,ab,kw OR randomised:ti,ab,kw OR randomly:ti,ab,kw OR phase:ti,ab,kw OR case:ti,ab,kw | 6608211 |
| #5 | #1 AND #2 AND #3 AND #4 | 219 |
| **Cochrane Library** | | |
| #1 | (lung cancer):ti,ab,kw OR (lung carcinoma):ti,ab,kw OR (lung neoplasm):ti,ab,kw OR (NSCLC):ti,ab,kw OR (lung adenocarcinoma):ti,ab,kw (Word variations have been searched) | 34686 |
| #2 | (epidermal growth factor receptor):ti,ab,kw AND (EGFR):ti,ab,kw (Word variations have been searched) | 1747 |
| #3 | (C797S):ti,ab,kw (Word variations have been searched) | 15 |
| #4 | (trial):ti,ab,kw OR (randomized):ti,ab,kw OR (phase):ti,ab,kw OR (case):ti,ab,kw OR (comment):ti,ab,kw (Word variations have been searched) | 1570136 |
| #5 | #1 AND #2 AND #3 AND #4 | 4 |
| **ClinicalTrials.gov** | | |
| #1 | (“lung cancer” OR “lung carcinoma” OR “lung neoplasm” OR “NSCLC” OR “lung adenocarcinoma”) AND (“epidermal growth factor receptor” OR EGFR) AND (“C797S”) AND (randomized OR randomised OR randomly OR random OR phase) | 11 |

* All literature published up to May 01, 2024.

**Figure S3. Study flowchart.**

Records identified from database searching

(n = 482)

Duplicate records removed

(n = 100)

**Research**

Review: 44

Cis C797S: 144

Other mutations: 31

Preclinical trial:43

Wrong methods:22

Duplicates: 74

No data: 12

Records screened

(n = 382)

Irrelevant records

(n = 370)

**Screening**

Excluded records

(n = 5)

No data: 2

Wrong methods: 2

Other mutations: 1

Full-text articles assessed for eligibility

(n=12)

(n =130)

**Eligibility**

Records from other sources

(n = 3)

**Included**

Studies included

(n =10)
